# South West Surgical Club—Meeting in Truro and Penzance

**Published:** 1988-08

**Authors:** 


### Surgical Club of South West England

### Meeting at Truro and Penzance May 1987

### MANAGEMENT OF MALIGNANT PLEURAL EFFUSION WITH TALC PLEURODESIS

Syed S. Hasan
Royal Cornwall Hospital, Truro

Pleural effusion is a common complication of advanced malignant disease, causing distressing symptoms. Simple aspiration results in reaccumulation of fluid. Effective local treatment which prevents recurrence and reduces symptoms is needed.

Forty patients with malignant effusion were treated with Iodised Talc Pleurodesis: these were fourteen males and twenty-six females, in an age group ranging from forty-two to sixty-seven years. The commonest primary malignancy being breast in twenty-five cases. Treatment was administered under general anaesthesia, with a single puncture thoracoscope, allowing complete aspiration of effusion, and pleural biopsy in each case. Pleurodesis was achieved using 1% Iodised Talc, sprayed uniformly under direct vision. Intercostal drainage was left in all cases.

There was one post-operative death. Follow-up was completed in thirty-nine patients up to a period of twelve months. Effusions recurred in two patients (5.1%) at three months. Complete symptomatic relief was achieved in twenty-nine patients (74%). Five patients had minimal symptoms; five patients had no symptomatic relief despite absence of the effusion. Local Talc Pleurodesis is a very effective method of treatment for malignant pleural effusion.

### 'IATROGENIC' URETHRAL STRICTURES

by Martin L. Crosfill, Nick J. Barwell
West Cornwall Hospital, Penzance

Having been faced with one or two cases of urethral strictures associated with catheterisation, the first speaker (MLC) presented a retrospective study of 37 stricture patients presenting over a seven year period. No clear cause could be found in 13 of the cases. Urological operations (mostly TUR) accounted for 9 and catheterisation (of ill patients for nursing convenience or for monitoring purposes) for eleven.

Long (shaft) or multiple strictures predominated in this group (10/11) but were absent from the urological trauma group (all short single strictures). It was concluded that catheter strictures formed a separate group which was 'inflammatory' in origin. The question was raised, does the act of catheterisation in an ill patient carry a risk of stricture formation or does the fault lie in particular catheters?

The second speaker (NJB) demonstrated a Silicone Latex 'Tinkler' (22 or 24 FG 3 way) catheter which had, when first purchased given rise to no problems but when a batch was used which had been stored for two or more years, 12 out of 19 patients who had undergone transurethral resections developed urethral strictures. In this instance at least, some aspect of the ageing, storage or manufacture of the catheters appeared to have caused the trouble.

### LOOKING BACK TO 1962

N. J. Barwell
Royal Cornwall Hospital, Truro

A comparison of workload of a House Surgeon at a London Teaching Hospital in 1962, with a Surgical team at Truro in 1967.

The Teaching Hospital team consisted of a Consultant, Senior Registrar, half the time of a Senior House Officer and a House Surgeon. On take 1 day in 4.

The Truro team, a Consultant, American Registrar at the equivalent of first year Registrar grade and two House Surgeons.

The workload was compared from discharge summaries examined retrospectively for 1962. This took about 20 hours. The analyses of the last 6 months required about 4 minutes of computer time.

The District Hospital had three times as many admissions, four times as many operations and of particular interest, the shortened length of stay and lessened complication rate. 24 cases of appendicitis used 103 bed days in Truro whilst 13 required 113 in London. Five cases of carcinoma of the colon and rectum in London stayed just over 30 days each. 18 cases in Truro, 11 days.

The Teaching Hospital firm in London had a special interest in colo-rectal surgery.

19 groin herniae occupied a total of 146 bed days in London, whereas, 130 in Truro required 338 nights in hospital.

It is appreciated that a retrospective survey is never as accurate as a prospective survey. Comparison of the increasing pressures and workload of a District Hospital compared with a Teaching Hospital, especially with less staff, will I hope lead to a better distribution of resources.

### ASPECTS OF NEAR DROWNING

A. D. Simcock
Royal Cornwall Hospital, Truro

Death from drowning is the third commonest cause of death in children and accounts for 9000 deaths per annum in the United States of America. Drowning occurs either from an inability to swim or unconsciousness. Hypothermia causes both and is the most significant factor in deaths amongst swimmers. Several medical conditions can cause loss of consciousness in the water.

Successful treatment depends on rapid assessment and relief of hypoxia. All patients who have had to be rescued should be admitted. Those who have inhaled should be monitored closely. If there is any doubt about the adequacy of ventilation, then intubation and mechanical ventilation should be undertaken without delay.

Care must be taken in assessing the apparently dead. Several children have recovered normally after prolonged submersion and long periods of cardiac arrest. Again, profound hypothermia can mimic cardiac arrest and the victim should not be abandoned before an adequate history has been sought, temperature measured, and an E.C.G. taken.

The Truro results show that patients who reach us before they have arrested have an excellent prognosis. We have two survivors from the cardiac arrest group, one recovered completely, but the second is still paraplegic.

### CUSTOMER SATISFACTION ON THE SURGICAL WARD

**J. F. L. Shaw**
**Derriford Hospital, Plymouth**

The small number of patients who complain about their hospital stay may not be truly representative of patient dissatisfaction, and the present study was developed to obtain further information about patient opinions.

In February and March 1987, 50 adult patients leaving our surgical ward were given a questionnaire to complete. This asked for a grading of from 1 to 10, where 1 is very bad and 10 is very good, of the following: Admission Procedure, Hospital Accommodation, Hospital Staff generally, Hospital Food, Hospital Organization, Treatment for the Illness, Information given about the Illness, Pain after the Operation. Patients were also asked to comment about the worst aspects of their stay, and how the hospital could be improved.

In the grading, grades below 5 appeared in only three categories: Food (8% below grade 5), Pain (6%), and Information (4%). Grade 10 was achieved in Treatment (88% grade 10), Staff (86%), Accommodation (76%), Admission Procedure (74%), Information (72%), Organization (62%), Food (38%) and Pain (28%).

The worst aspects of stay were uncomfortable beds (10%), boredom (8%) and smoking in the dayrooms (6%).

### "NEW APPROACHES TO THE MANAGEMENT OF ADVANCED" PROSTATIC CANCER

**D. A. Gillatt, J. C. Gingell**
**Southmead Hospital, Bristol**

Prostatic carcinoma is the second most common malignancy in men over 60 years of age, and there is much renewed research interest in the condition. New modalities allow for earlier diagnosis of the condition and better monitoring of the clinical course. Transrectal ultrasound scanning, isotope bone scanning and serum prostate specific antigen measurements allow accurate assessment of the disease.

Local malignancy confined to the prostate gland is often managed conservatively. External beam radiotherapy and interstitial Iodine 125 seeds are both being used in Bristol and other centres to treat local disease. Evidence is mounting in favour of radical prostatectomy as a suitable alternative and this method of treatment may well become more prevalent.

Once the carcinoma has spread beyond the prostate endocrine manipulation is usually employed. Androgen ablation either by bilateral orchidectomy or with oestrogens have traditionally been used though both have their problems, the former psychological and the later cardiovascular.

Depot LH RH agonists are being assessed in Bristol as are antiandrogens such as cyproterone acetate. The modes of action of these agents varies but similar response rates and survival times are achieved. Other authors have suggested a significant improvement in results if adrenal as well as testicular androgens are suppressed. A large multicentre trial is underway to see if an improved response can be achieved by combining LH RH agonists with antiandrogens, thus achieving total androgen ablation. All these new treatments are expensive and the cost must be weighed against any clinical benefit from their use.

### RETRIEVAL OF ORGANS FOR TRANSPLANTATION IN CORNWALL 1986

**Giles Morgan**
**Royal Cornwall Hospital, Cornwall**

During 1986 donorship operations in the South Western Region yielded 101 kidneys, 9 livers, 11 hearts and 2 heart lung specimens for transplantation.

Retrospective analysis of the mortality statistics from the Intensive Care Unit at the Royal Cornwall Hospital Treliske was undertaken to assess the contribution made to organ donorship. During 1986 the 4-bedded Intensive Care Unit at RCH Treliske dealt with 277 patients of which 34 (12.3%) died. Only 3 patients fulfilled the criteria for organ donation specified by transplanting hospitals and organs were donated from 2 of these (kidneys 2, livers 2, heart 1). Permission for donorship was not requested from the third suitable patient because of events surrounding the patient's death. A voluntary offer of organ donorship was offered in one of the 34 deaths but was refused on the basis of old age. No donors carried a donor card.

The problems of organ donorship in a District General Hospital were summarised and included (a) unfamiliarity with the procedure which would be expected to take place 3 or 4 times per year; (b) failure of prospective donors to fulfil requirements because of death before adequate resuscitation, episodes of profound hypotension, sepsis and old age related diseases such as peripheral vascular disease and neoplasia; and (c) inadequate education of the general public and medical profession in the benefits of organ donation.

The procedure for organisation of organ donation at RCH Treliske was described with particular attention to a computer generated flow chart describing the steps involved from the diagnosis of brain stem death to the departure of visiting surgical teams. The procedure was supervised by a member of the Intensive Care Consultant staff and updated after each donor procedure. The use of the protocol had simplified many of the organisational difficulties previously encountered but it was noted that from beginning to end each donorship procedure took between 12 and 18 hours to organise and carry out.

### COMPUTERIZED SURGICAL AUDIT—GETTING THE SYSTEM AIRBORNE

**John Rickett**
**Torbay Hospital, Torquay**

Increasing pressure to justify spending in surgical units by general managers combined with increasing demands to provide details of workloads, throughput, results and complications has prompted some software firms to produce surgical audit systems with the ability to search all aspects of surgical work.

For those working in hospitals it is clear the hospital activity analysis is inadequate in many respects. Not only is the diagnostic input inaccurate and incomplete, but also it is very difficult to get the information out.

In applying for funds for setting up an audit system a major consideration is whether staff will be required to work it. Choice of software is dependent on this. Therefore the ability to generate discharge summaries is a useful facility so that secretarial time is effectively saved.

Many software firms are in financial difficulty and obviously it is most important to patronize an established firm in order to avoid the risk of the software getting out of date soon after it has been installed. The contract to service and update the software is of paramount importance. However carefully the system is designed prior to installation there invariably will be necessary modifications as surgical patterns change, new methods introduced and work patterns modified to suit each surgical unit. At the present time the cost of setting up an audit system is not prohibitively expensive, and should be seriously considered by all with more than ten years professional life ahead.

### COMPUTERS IN SURGERY: RESULTS ILLUSTRATE THE NEED

**R. J. Lawrence**
**Torbay Hospital, Torquay**

A computerised audit system has been available in one Surgical unit; March 1987 signalled the first year of its use experimentally. During this period 1307 admissions to 26 beds (50.25 pt/bed/y) occurred. 571 (43%) were admitted as emergencies. 576 (44%) were aged over 60y and 311 (23%) aged under 30y. Operative treatment was required on 1063 (83%) of admissions and of these 132 (12.4%) suffered a total of 177 individually categorised post-operative complications (rate of 16.7%) 65 patients (6.1%) died within one month of operation and of those admitted as an emergency 8.1% died. 82% were over the age of 70y.

This presentation of basic information is intended to illustrate base data retrieval using the micro-computer. Indepth analysis is available e.g. specific nature of complications, related to age, pathological process including pre- and post-operative factors, plus the effect on duration of hospital stay. Personal operation and complication files and with long-term usage analysis of trends, monthly and yearly become available.

The provision of accurate information of clinical activity retrieved easily and quickly we find interesting, stimulating, increases efficiency within the unit, with the ultimate aim of providing a better service and enhancing quality of patient care.

